# Neurophysiological markers predicting recovery of standing in humans with chronic motor complete spinal cord injury

**DOI:** 10.1038/s41598-019-50938-y

**Published:** 2019-10-09

**Authors:** Samineh Mesbah, Federica Gonnelli, Claudia A. Angeli, Ayman El-baz, Susan J. Harkema, Enrico Rejc

**Affiliations:** 1 Kentucky Spinal Cord Injury Research Center, University of Louisville, Louisville, Kentucky USA; 2 Department of Electrical and Computer Engineering, University of Louisville, Louisville, Kentucky USA; 3 Department of Bioengineering, University of Louisville, Louisville, Kentucky USA; 4 Department of Medicine, University of Udine, Udine, Italy; 5 School of Sport Sciences, University of Udine, Udine, Italy; 6 Frazier Rehab Institute, Kentucky One Health, Louisville, Kentucky USA; 7 Department of Neurological Surgery, University of Louisville, Louisville, Kentucky USA

**Keywords:** Diseases of the nervous system, Spinal cord

## Abstract

The appropriate selection of individual-specific spinal cord epidural stimulation (scES) parameters is crucial to re-enable independent standing with self-assistance for balance in individuals with chronic, motor complete spinal cord injury, which is a key achievement toward the recovery of functional mobility. To date, there are no available algorithms that contribute to the selection of scES parameters for facilitating standing in this population. Here, we introduce a novel framework for EMG data processing that implements spectral analysis by continuous wavelet transform and machine learning methods for characterizing epidural stimulation-promoted EMG activity resulting in independent standing. Analysis of standing data collected from eleven motor complete research participants revealed that independent standing was promoted by EMG activity characterized by lower median frequency, lower variability of median frequency, lower variability of activation pattern, lower variability of instantaneous maximum power, and higher total power. Additionally, the high classification accuracy of assisted and independent standing allowed the development of a prediction algorithm that can provide feedback on the effectiveness of muscle-specific activation for standing promoted by the tested scES parameters. This framework can support researchers and clinicians during the process of selection of epidural stimulation parameters for standing motor rehabilitation.

## Introduction

Individuals with motor complete spinal cord injury (SCI) are unable to stand, walk, or move their lower limbs voluntarily; this condition drastically affects their quality of life and implies severe limitations for functional recovery^1,2^. In the last years, there has been increasing evidence that the combination of lumbosacral spinal cord epidural stimulation (scES) and activity-based training that includes standing and stepping practice can promote the recovery of standing, walking and volitional leg movements in chronic, clinically motor complete^3–7^ and incomplete^8^ SCI individuals. To date, the prevailing view is that scES modulates the excitability of lumbosacral spinal circuitry by recruiting afferent fibers carrying somatosensory information^9–12^. This excitability modulation, in turn, can enable the spinal circuitry to generate appropriate muscle activation patterns in response to sensory information^5,13^, and can also allow residual functionally silent descending input to modulate standing and stepping activation patterns^3,4,7,14^.

The ability to stand with independent lower limb extension is a key achievement toward the recovery of functional mobility, and was consistently observed in all three motor complete SCI individuals that subsequently recovered over ground stepping and walking^3,7^. We showed that the appropriate selection of individual-specific scES parameters is crucial to promote standing with independent lower limb extension in this population^13^. The guidelines proposed for selecting a sub-set of electrode configurations to be tested for facilitating standing include adjusting cathodes (active electrodes) positon in order to target primarily extensor muscle groups according to the individualized map of motor pools activation^13^. Also, the use of multiple interleaving programs represents an important advantage compared to the use of a single program, as it allows to access different locations of the spinal circuitry with different intensities. However, to date there are no available algorithms or procedures that suggest the exact set of parameters to be applied for facilitating standing using tonic scES. In addition, the characteristics of muscle activation patterns leading to independent standing remain poorly understood. We have observed that EMG patterns that alternate bursts and negligible activation (i.e. similar to a rhythmic pattern) always result in poor standing ability^6^. On the other hand, overall continuous (i.e. non-rhythmic) co-activation of several lower limb muscles can promote standing with independent lower limb extension^6,7^, but can also be observed during assisted standing. Understanding the characteristics of muscle activation patterns leading to independent standing can be of great importance for developing machine learning models capable of contributing to the selection of appropriate scES parameters.

Here, we introduce a novel framework for EMG data processing that implements spectral analysis and machine learning methods for characterizing EMG activity resulting in independent or assisted standing, and for identifying which of the tested sets of stimulation parameters promote muscle activation more effective for standing. We initially determined which spectral analysis method is more effective for identifying frequency-domain EMG features that characterize independent standing promoted by scES in humans with clinically motor complete SCI. We then integrated EMG frequency- and time-domain features in the computational model and tested its ability to accurately classify independent and assisted standing. Also, the physiological characteristics of EMG activity resulting in assisted and independent standing were defined. Finally, we applied the proposed framework on EMG datasets collected while research participants were testing different scES stimulation parameters for standing in order to rank the effectiveness of the muscle activation generated.

## Results

### Standing motor patterns with and without scES

**Figure 1:**
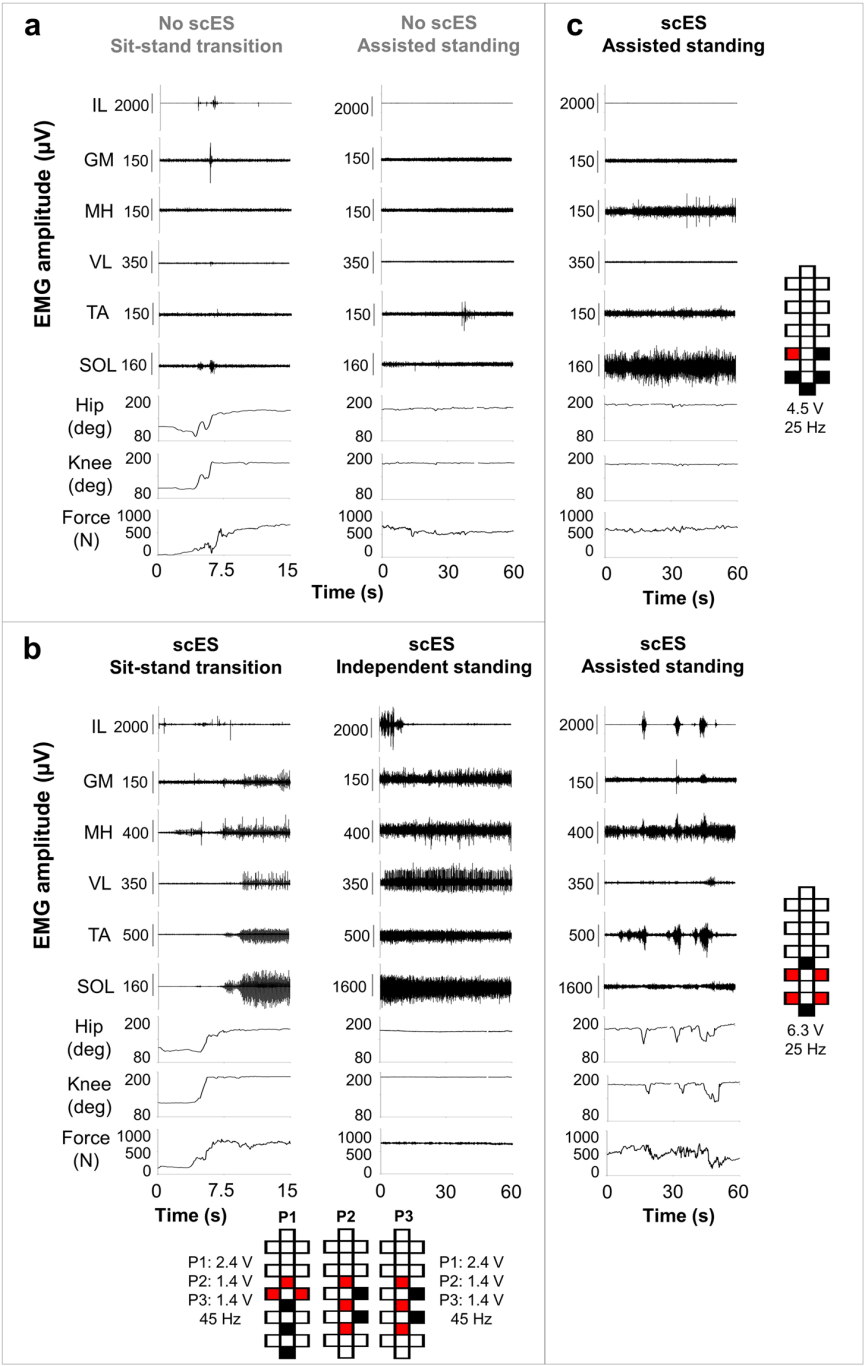
EMG, lower limb joint angles and ground reaction forces during sit-to-stand transition and during standing. Electromyography (EMG), hip and knee joint angle, and ground reaction forces recorded from research participant A59 during: (**a)** sit-to-stand transition and standing with external assistance for hips and knees extension (assisted standing) without spinal cord epidural stimulation (scES); (**b)** sit-to-stand transition and independent standing using scES; the participant held the hands of a trainer for balance control; **(c)** assisted standing with scES resulting from an overall continuous activation pattern (top) and from an EMG pattern characterized by the alternation of EMG bursts and little activation (bottom). Stimulation amplitude, frequency and electrode configuration (cathodes in black, anodes in red, and inactive in white) are reported for each standing condition. In (**b)**, the participant was stimulated with 3 programs delivered sequentially at 15 Hz, resulting in an ongoing 45 Hz stimulation frequency. EMG was recorded from the following muscles of the right lower limb: IL, iliopsoas; GL, gluteus maximus; MH, medial hamstring; VL, vastus lateralis; TA, tibialis anterior; SOL, soleus.

Research participants required external assistance for lower limb extension when scES was not provided (Supplemental Video 1). Limited EMG activity was generally observed in response to the assisted sit-to-stand transition, and negligible EMG was recorded during standing with external assistance for hips and knees extension (assisted standing; Fig. 1a). When scES parameters for standing were applied, little activity and no movement was directly induced in sitting (Fig. 1b). On the other hand, without any change in stimulation parameters, sensory information related to the sit-to-stand transition and loading of the legs resulted in the generation of motor patterns with different characteristics (Fig. 1b,c; Supplemental Video 1 and 2). We have consistently observed that standing with independence of hip and knee extension (independent standing) is enabled by overall continuous (i.e. non-rhythmic) EMG activity of primary lower limb muscles crossing the hip, knee and ankle joints, with the exception of iliopsoas (Fig. 1b). However, continuous EMG patterns in these muscles can also be insufficient for achieving independent standing (Fig. 1c, top). On the other hand, the alternation between EMG bursts and negligible activity (i.e. similar to a rhythmic pattern) in lower limb muscles always resulted in assisted standing (Fig. 1c, bottom).

### Time- and frequency-domain EMG features can accurately classify assisted versus independent standing

**Figure 2:**
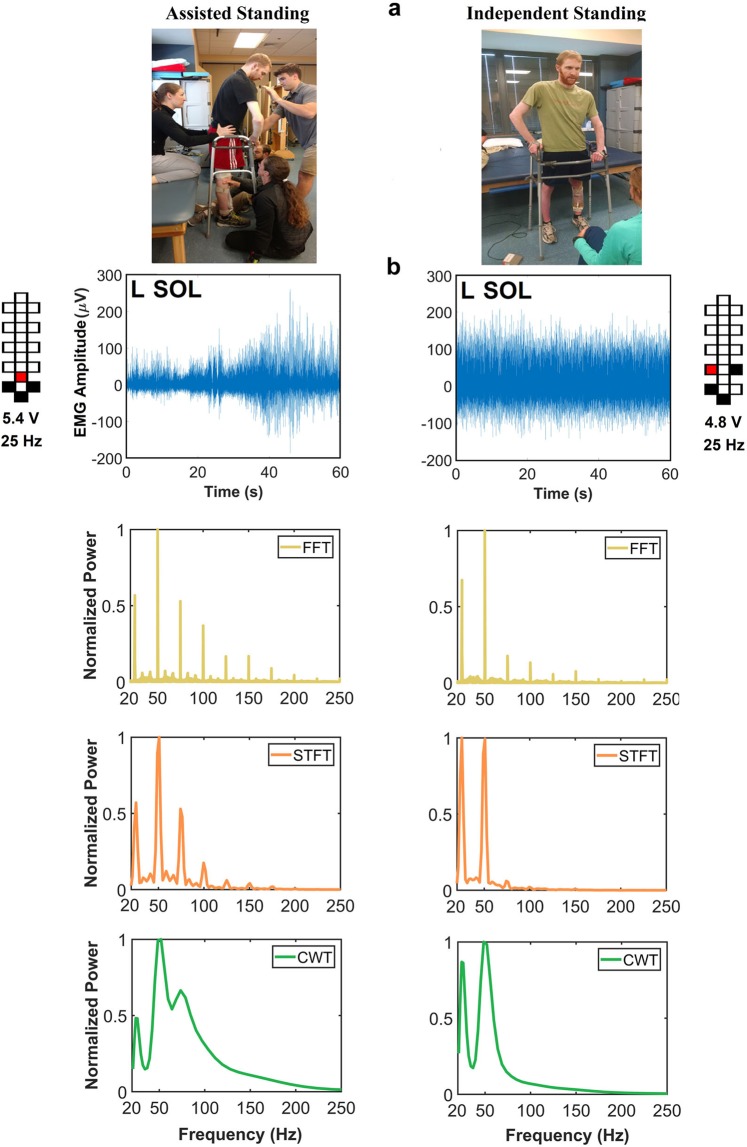
Spectral power density of EMG collected during standing. (**a)** Exemplary images of assisted standing (left) and independent standing (right). (**b)** EMG activity recorded from the left soleus (L SOL) of research participant A45 during assisted standing (left) and independent standing (right) with epidural stimulation, and related spectral power density generated by Fast Fourier Transform (FFT), Short-Time Fourier Transform (STFT) and Continuous Wavelet Transform (CWT). Stimulation amplitude, frequency and electrode configuration (cathodes in black, anodes in red, and inactive in white) are reported.

Two time-domain EMG variables (total power and pattern variability) were initially included in the proposed data processing framework aimed at classifying assisted and independent standing. This approach led to a classification accuracy for assisted and independent standing equal to 83.7% when all investigated muscles were considered for analysis. To improve this classification accuracy, we explored the inclusion of frequency-domain EMG features in the computational model. An initial step was devoted to the selection of an effective analysis method for EMG activity promoted by scES. When exemplary EMG signals recorded during assisted and independent standing were considered for analysis (Fig. 2a), Fast Fourier Transform (FFT) and, to a less extent, Short-Time Fourier Transform (STFT) primarily highlighted the content of frequencies related to epidural stimulation frequency (25 Hz) and its harmonics (Fig. 2b). On the other hand, Continuous Wavelet Transform (CWT) showed relevant frequency content that was not related to scES frequency. Also, the power of EMG signal collected during independent standing tended to be shifted toward lower frequency bins compared to that recorded during assisted standing. We then applied these three signal analysis methods on all EMG data collected during assisted and independent standing events from the 11 research participants considered in this study (Supplemental Table 1).

**Figure 3:**
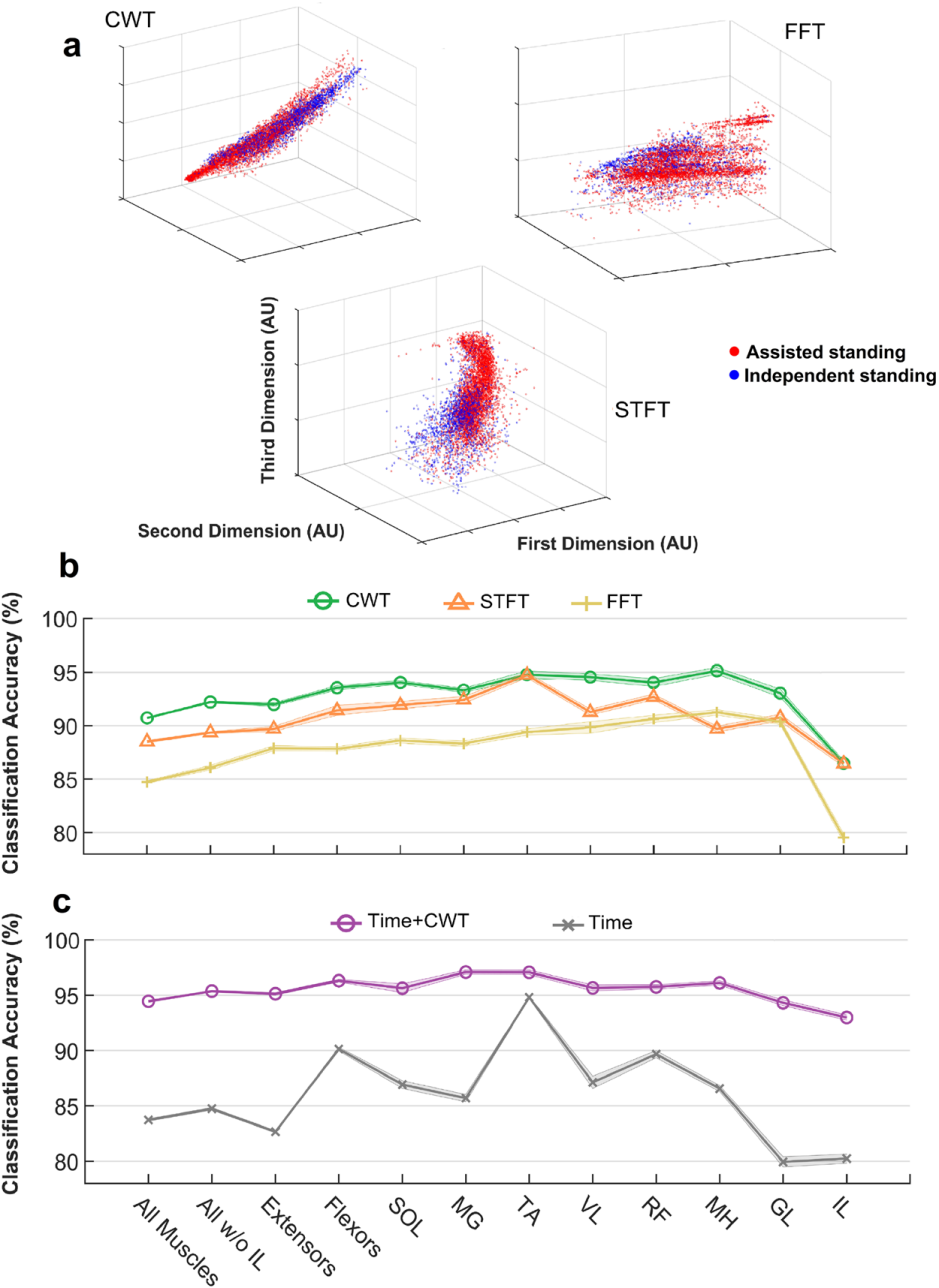
EMG-based classification of assisted vs independent standing. (**a**) First three dimensions of EMG standing data points (blue: independent standing, n = 2032 (127 standing events * 16 muscles); red: assisted standing, n = 4984 (316 standing events * 16 muscles, excluding 72 data points because of technical issues during EMG recordings) after normalization, dimension reduction and logarithmically transforming the spectral feature vectors extracted from Continuous Wavelet Transform (CWT), Short-Time Fourier Transform (STFT) and Fast Fourier Transform (FFT). Independent standing: independent lower limb extension and self-assistance for balance; assisted standing: external assistance for hip and knee extension and self-assistance for balance. K-nearest neighbor classification accuracy of the standing condition (assisted or independent standing) provided by feature vectors extracted from CWT, STFT and FFT **(b)**, and by time-domain features only or by the integration of time-domain features and CTW-extracted features (**c)**, when considering all investigated muscles (left and right soleus (SOL), medial gastrocnemius (MG), vastus lateralis (VL), rectus femoris (RF), gluteus maximus (GL), tibialis anterior (TA), medial hamstring (MH), iliopsoas (IL); all muscles except left and right IL; primary extensor muscles (left and right SOL, MG, VL, RF, GL); primary flexor muscles (left and right TA and MH); and each investigated pair of muscles separately (left and right SOL, MG, TA, VL, RF, MH, GL, IL).

After normalization, dimension reduction and logarithmically transforming the EMG spectral feature values, the first three dimensions of standing data points (blue: independent standing; red: assisted standing) derived from the tested spectral analysis methods were plotted in Fig. 3a. It can be noted that the three analysis methods result in different distributions of the data points, and that CWT tends to present a clearer visual discrimination between assisted and independent standing data points. These feature values were subsequently used as input for K-nearest neighbor (KNN) classification. As expected from the exemplary data analysis and from the data points presented in Fig. 3a, we observed that CWT-derived features promoted the highest classification accuracy for assisted versus independent standing compared to STFT- and particularly FFT-derived features (Fig. 3b).

Hence, CWT-derived data were integrated with time-domain EMG features (EMG total power and pattern variability), resulting in a classification accuracy for assisted versus independent standing ranging from 92.2% to 97.5%, depending on the considered muscle(s) (Fig. 3c). This classification accuracy is higher and more consistent across examined muscles compared to when either frequency- or particularly time-domain EMG features alone were considered (Fig. 3b,c). Based on the results reported in this section, CWT-derived data were also considered for further analysis aimed at describing the physiological characteristics of muscle activation during standing with scES.

### Physiological characteristics of muscle activation resulting in assisted or independent standing

**Figure 4:**
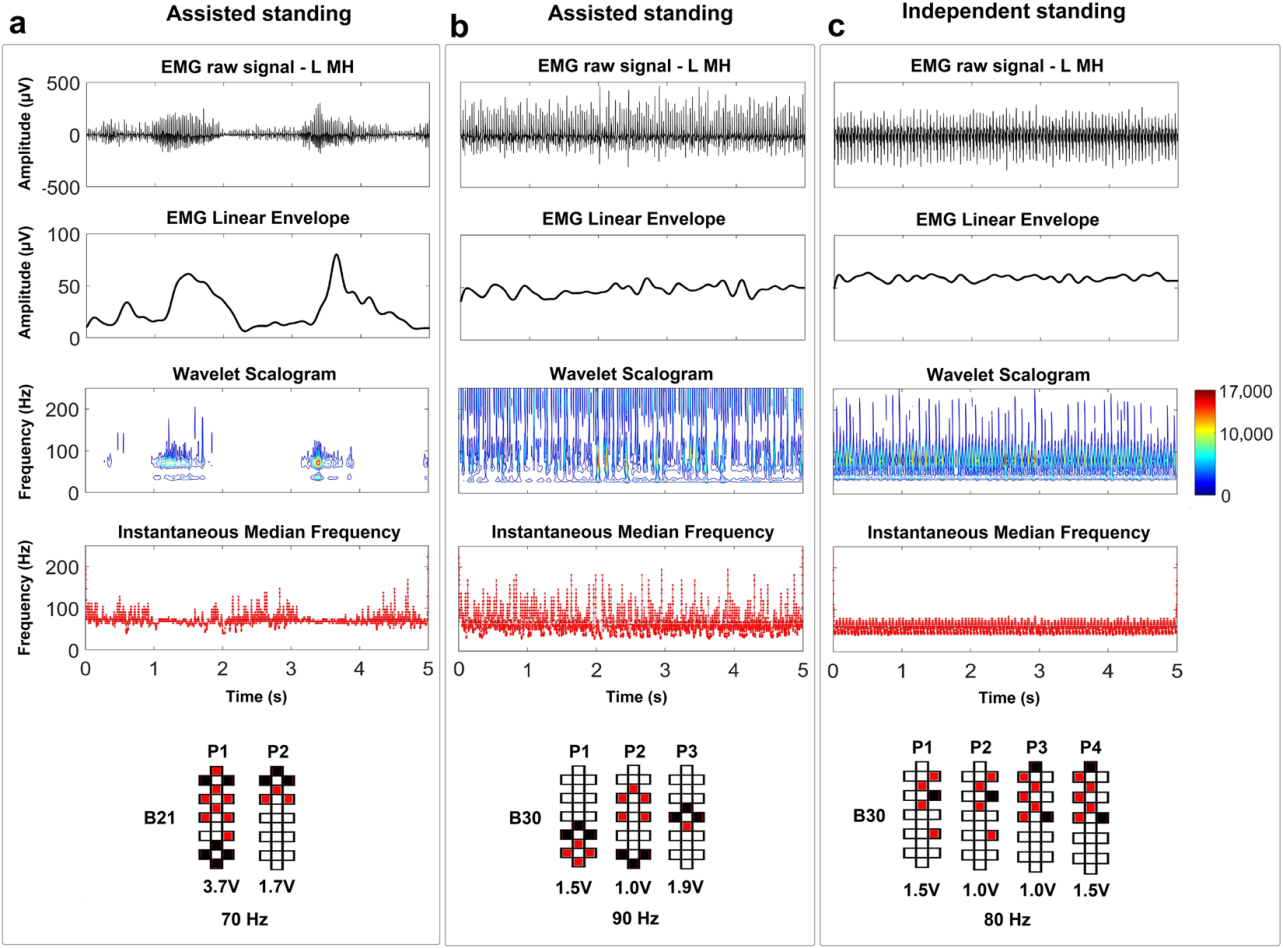
EMG-time and –frequency features characterizing exemplary standing activation patterns. EMG activity recorded from the left medial hamstrings (L MH) during standing with self-assistance for balance and external assistance for hip and knee extension characterized by (**a**) EMG bursts or by (**b)** continuous EMG activity, as well as (**c**) during independent standing with self-assistance for balance. For each standing condition, the EMG linear envelope, time-frequency power distribution of the signal (wavelet scalogram), and instantaneous median frequency are calculated from the plotted raw EMG. The wavelet scalogram is presented as contour plots, the power values of which are represented as colormaps, with the color scale showing the range of power values. Stimulation amplitude, frequency and electrode configuration (cathodes in black, anodes in red, and inactive in white) are reported. EMG activity reported in Panel A was collected from research participant B21. EMG activity reported in Panels B and C was collected from research participant B30.

Higher values of EMG pattern variability calculated from EMG linear envelope can characterize the muscle activation pattern consisting in the alternation between EMG bursts and lower activity (Fig. 4a; pattern variability = 0.68), which results in poor, assisted standing. On the other hand, this feature does not discriminate between overall continuous EMG patterns resulting in assisted standing (Fig. 4b; pattern variability = 0.23) or independent standing (Fig. 4c; pattern variability = 0.22). CWT can provide additional information based on instantaneous EMG time- and frequency-domain features. For example, in case of the alternation between EMG bursts and limited activation, the maximum power variability is also relevant (Fig. 4a, Wavelet Scalogram; EMG maximum power variability = 1.35) compared to the condition of assisted standing with continuous EMG pattern (Fig. 4b; EMG maximum power variability = 0.35).

It is worth noting that differences in the CWT pattern can be observed also between the two similar continuous raw EMG activity recorded from the same individual during assisted and independent standing (Fig. 4b,c, respectively). In particular, assisted standing (Fig. 4b) tended to present greater EMG maximum power variability (0.48), higher median frequency (70 Hz) and greater variability of median frequency (median frequency standard deviation = 32 Hz) compared to EMG activity that resulted in independent standing (0.35, 59 Hz, and 20 Hz, respectively; Fig. 4c). Interestingly, the same trend characterizing the differences in EMG features between assisted and independent standing can be also observed when the same exact stimulation parameters are applied (at different time points) to the same individual (Supplemental Fig. 1). In fact, even in this exemplary data, assisted standing tended to present greater EMG pattern variability (0.52), maximum power variability (1.51), median frequency (109 Hz) and median frequency standard deviation (49 Hz) as compared to standing with independent knees extension (0.23, 0.65, 81 Hz, and 23 Hz, respectively).

**Figure 5:**
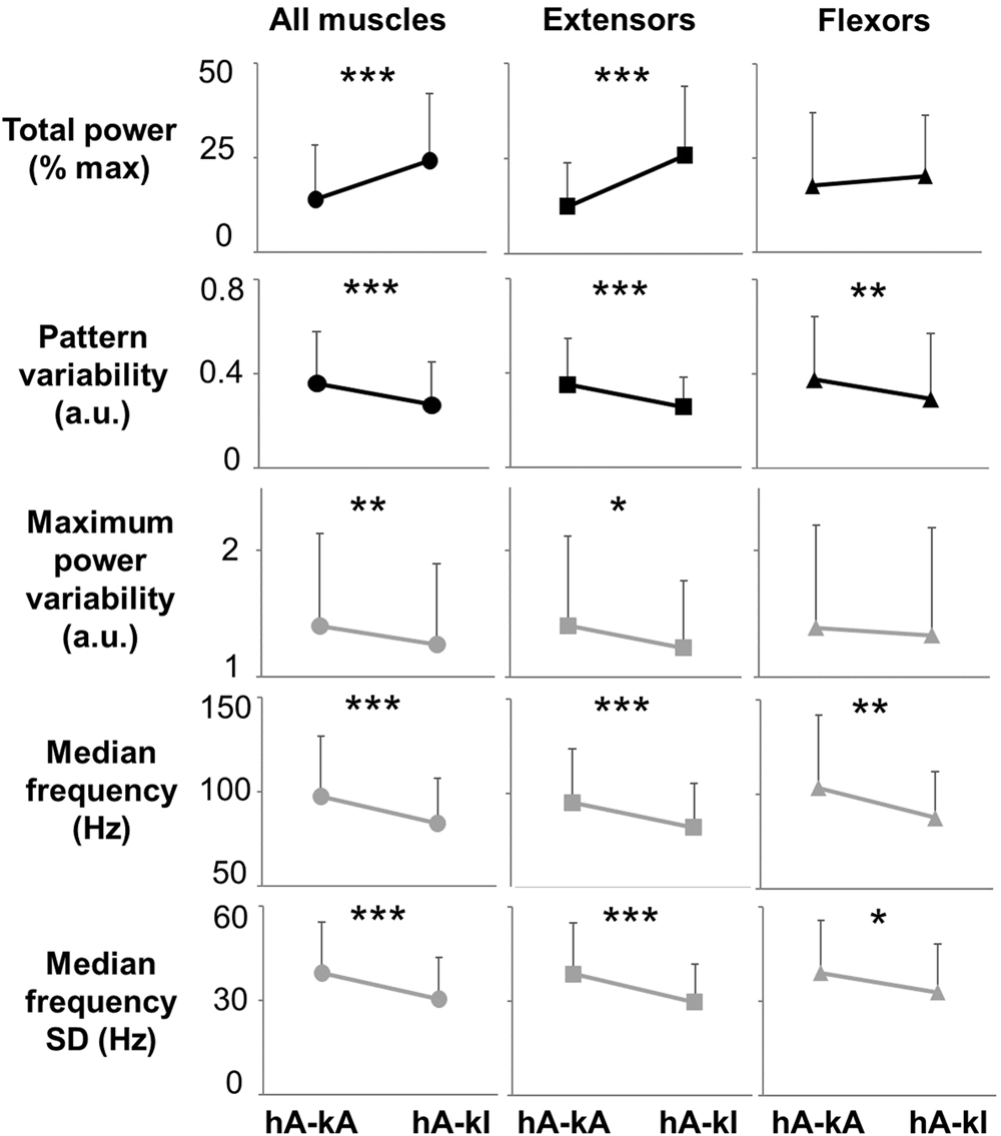
Quantification of EMG time- and frequency-domain features collected during standing with knees assisted or standing with independent knees extension. Representative time- and frequency-domain EMG features collected during standing with external assistance for hips and knees extension (hA-kA), and during standing with hips assisted and independent knees extension (hA-kI). EMG features values were averaged among research participants (n = 8) and among all investigated muscles (left and right soleus, medial gastrocnemius, vastus lateralis, rectus femoris, gluteus maximus, tibialis anterior and medial hamstring; total n = 114), among primary extensor muscles (left and right soleus, medial gastrocnemius, vastus lateralis, rectus femoris, gluteus maximus; total n = 80), or among primary flexor muscles (left and right tibialis anterior and medial hamstring; total n = 32). Values are expressed as mean ± standard deviation (SD). Differences were tested by Wilcoxon test. *p < 0.05; **p < 0.01; ***p < 0.001.

Paired comparisons (n = 8) show that standing with independent knees extension was promoted by significantly higher EMG total power, lower pattern variability, lower maximum power variability, lower median frequency standard deviation (SD), and lower median frequency as compared to assisted standing (Fig. 5). These differences were more pronounced when all investigated muscles and primary extensor muscles were considered as compared to primary flexor muscles. It is also worth noting that the average stimulation frequency was similar between the two conditions (47 ± 24 Hz for assisted standing and 51 ± 27 Hz for independent knees extension; p = 0.844).

We then performed a similar comparison including the 5 individuals who achieved assisted standing, standing with external assistance at the hips and independent knees extension, and independent standing (Supplemental Fig. 2). In summary, no substantial differences were observed between standing conditions with hips assisted or hips independent while the knees achieved independent extension. On the other hand, these two standing conditions demonstrating independent knees extension were characterized by higher EMG total power, lower pattern variability, lower median frequency variability, and lower median frequency compared to standing with hips and knees assisted, showing the same trend already reported in Fig. 5. Also, the average stimulation frequency was similar across these three standing conditions (58 ± 24 in assisted standing; 61 ± 29 Hz in hips assisted and knees independent; 62 ± 33 Hz in independent standing; p = 0.182).

**Figure 6:**
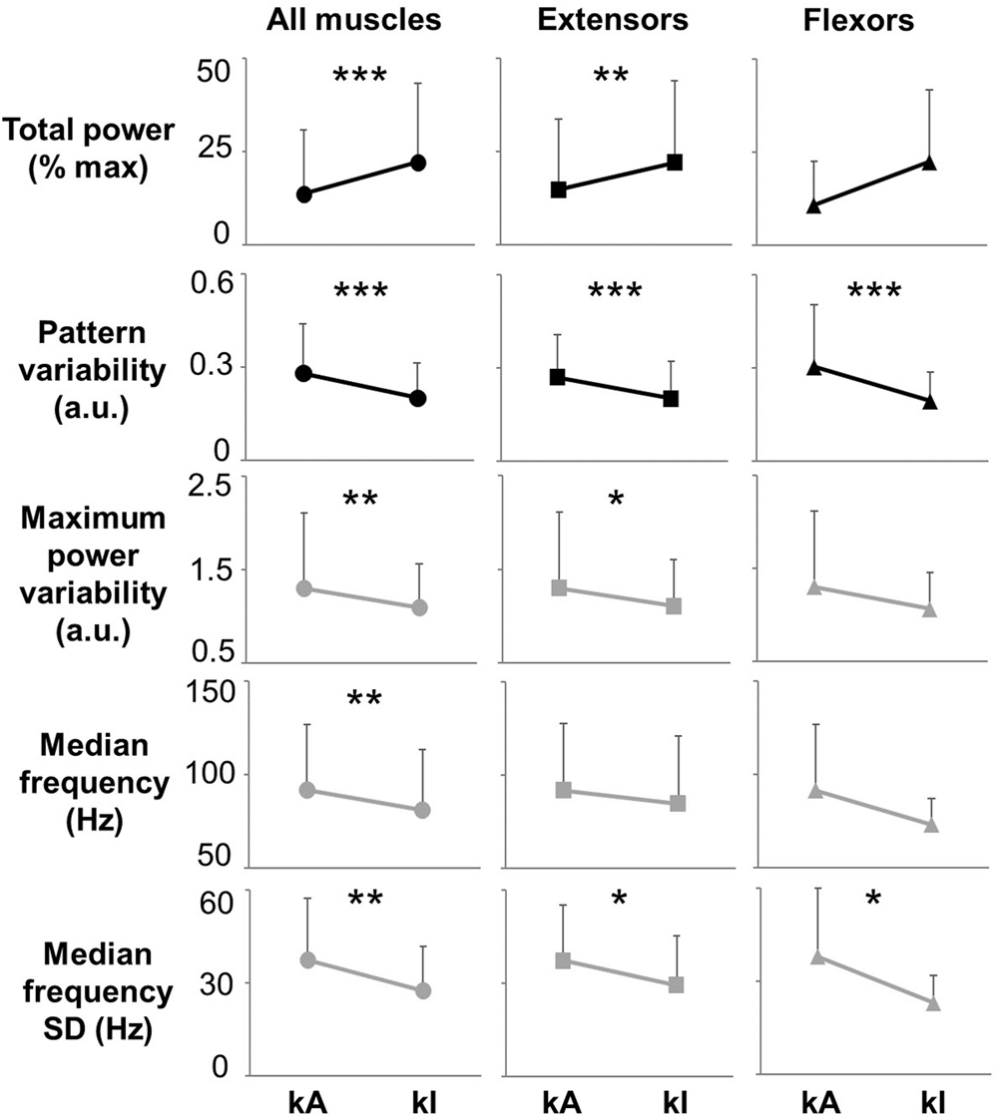
Quantification of EMG-time and –frequency domain features collected during standing with one lower limb assisted for knee extension. Representative time- and frequency-domain EMG features collected during standing when one lower limb achieved independent extension (kI) while the contralateral limb required external assistance for knee extension (kA). EMG features values were averaged among research participants (n = 7) and among different muscle groups of the assisted lower limb (kA) or the independent lower limb (kI). In particular, all investigated muscles (soleus, medial gastrocnemius, vastus lateralis, rectus femoris, gluteus maximus, tibialis anterior and medial hamstring; total n = 49), primary extensor muscles (left and right soleus, medial gastrocnemius, vastus lateralis, rectus femoris, gluteus maximus; total n = 35), or primary flexor muscles (left and right tibialis anterior and medial hamstring; total n = 20) were considered for analysis. Values are expressed as mean ± standard deviation (SD). Differences were tested by Wilcoxon test. *p < 0.05; **p < 0.01; ***p < 0.001.

We then assessed standing conditions during which one lower limb (i.e. left side) achieved independent extension while the contralateral lower limb (i.e. right side) required external assistance. Similarly to the previous findings, higher EMG total power, lower pattern variability, lower maximum power variability, lower median frequency SD, and lower median frequency were detected from the limb achieving independent extension (Fig. 6). This trend showed more consistent statistical significance when all investigated muscles were pooled together for analysis.

**Figure 7:**
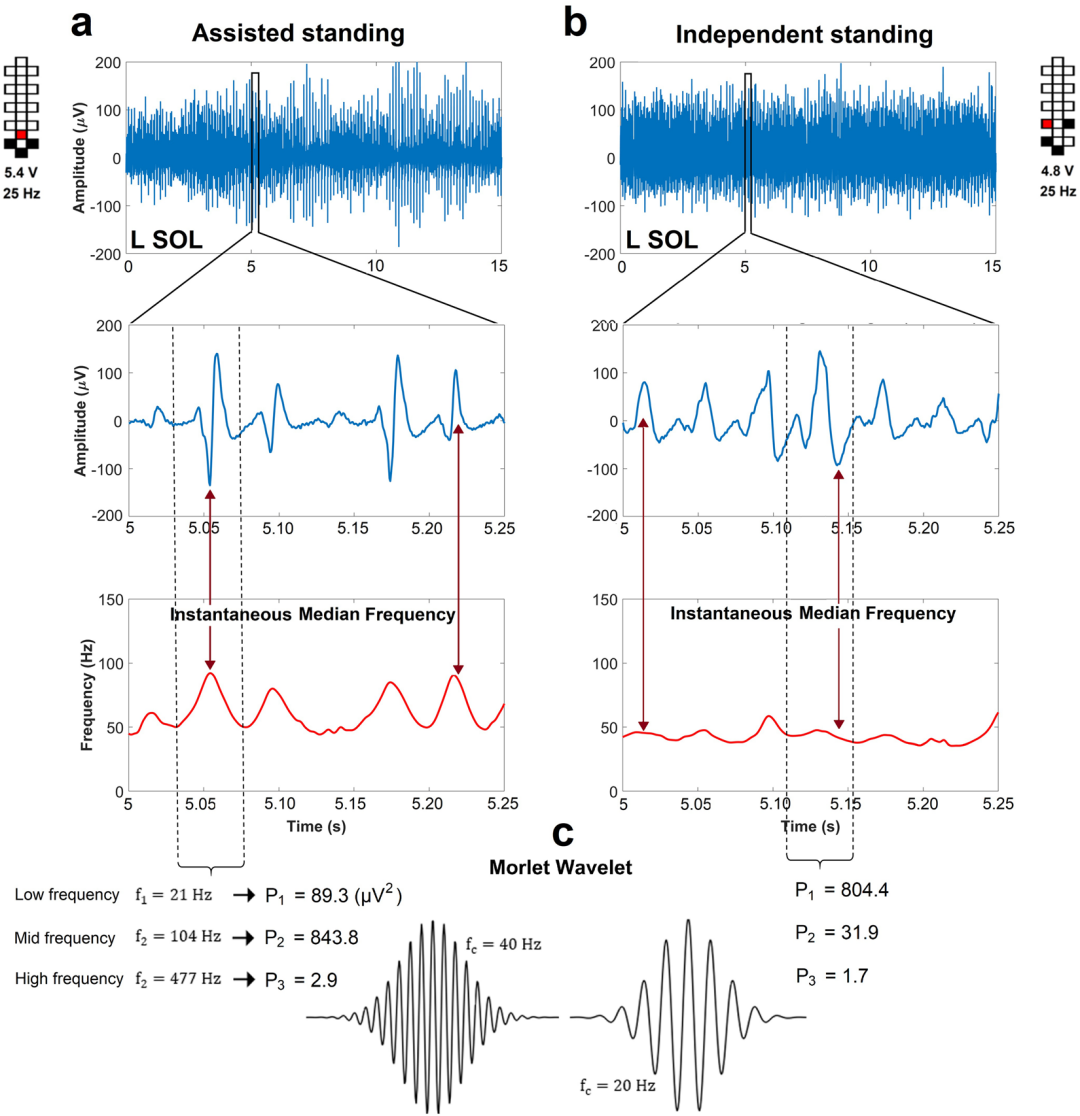
Representative spinal cord evoked responses collected during assisted and independent standing. EMG activity collected from the left soleus (L SOL) muscle of research participant A45 (**a)** during standing with external assistance for hips and knees extension and (**b)** during standing with independent lower limb extension. Spinal cord evoked responses and instantaneous median frequency calculated by continuous wavelet transform are reported for EMG activity included in the windows entered in the top panels. (**c)** Exemplary Morlet wavelet signals with high (40 Hz, left) and low (20 Hz, right) central frequency (f_c_). The signal power (P, µV^2^) at low (21 Hz), mid (104 Hz) and high (477 Hz) frequency bins is calculated for the spinal cord evoked responses highlighted by the dashed lines. Signal power collected during assisted standing is more concentrated at the mid frequency bin, while signal power collected during independent standing is more concentrated at the low frequency bin. Stimulation amplitude, frequency and electrode configuration (cathodes in black, anodes in red, and inactive in white) are reported for the two standing conditions.

It is worth noting that the higher median frequency and median frequency SD values observed during assisted standing can be attributed, at least partially, to the sharper peak shape of the spinal cord evoked responses generated (Fig. 7), which results in relevant increments of the instantaneous median frequency. Conversely, the smoother peaks of spinal cord evoked responses detected during independent standing contain more power at lower frequencies and result in a smaller instantaneous median frequency modulation.

### Ranking the effectiveness of EMG activity for standing

The high classification accuracy for assisted versus independent standing provided by our EMG-based framework (Fig. 3) led us to develop a further computational step (prediction algorithm) aimed at ranking the effectiveness of muscle activation patterns generated for standing. We initially trained muscle-specific KNN models based on three different standing data sets related to different external assistance for standing. Classification accuracy was high (95.3% on average) for the KNN model trained with assisted and independent standing data set (Supplemental Fig. 3), while lower accuracy was observed for the other two models (Supplemental Table 2).

**Figure 8:**
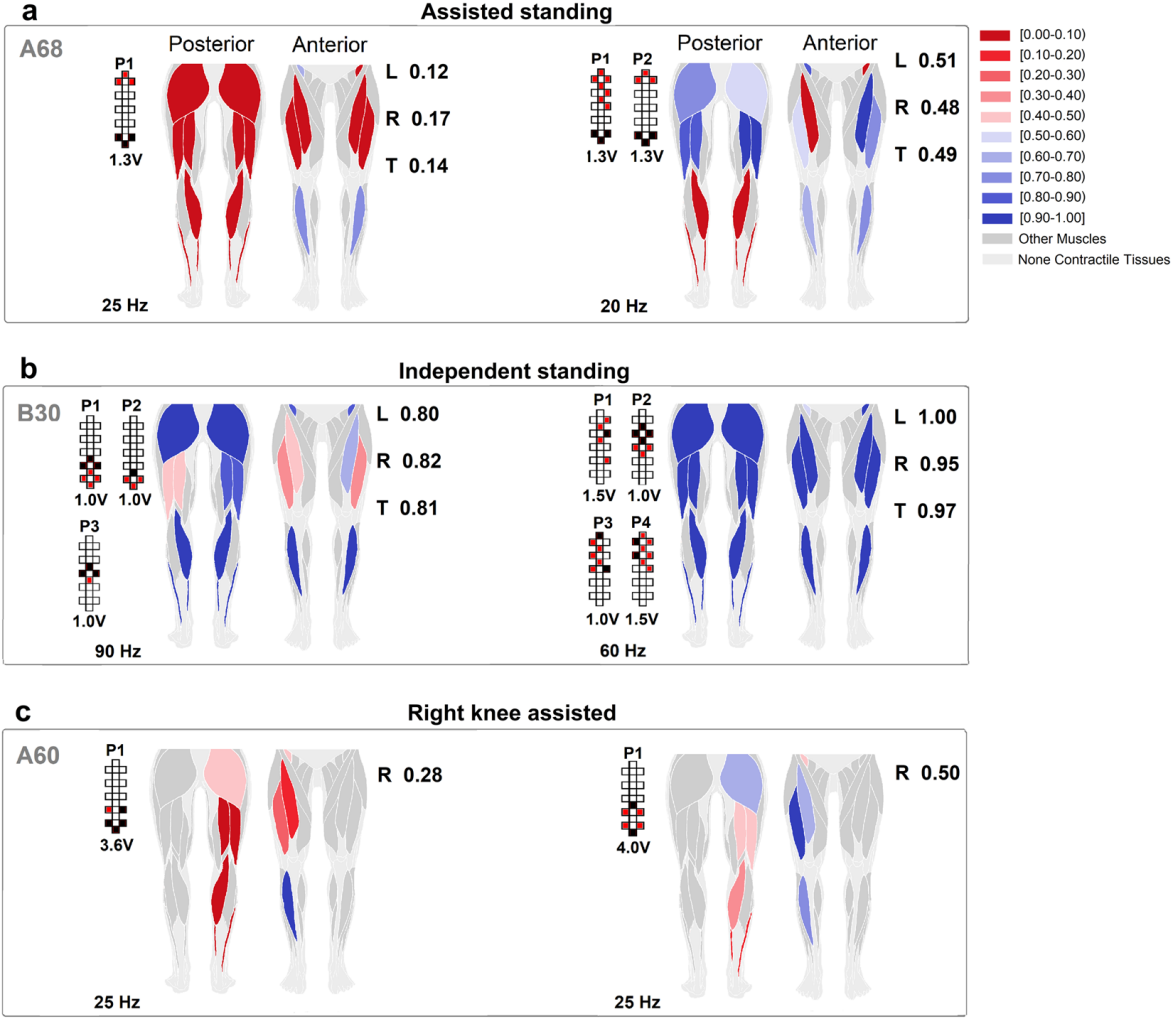
Colormap representing the effectiveness of standing muscle activation. The effectiveness of standing muscle activation is ranked by the prediction algorithm (see Methods). For each investigated muscle represented in the anatomical schematics, shades of red color rank activations labeled as assisted standing, while shades of blue color rank activations labeled as independent standing. Exemplary effects of different epidural stimulation parameters on muscle activation ranking during: (**a)** standing with external assistance for hips and knees extension; (**b)** standing with independent lower limbs extension; (**c**) standing with one lower limb assisted (right side) while the contralateral limb achieved independent extension. Average ranking score for the investigated muscles of the left (L) and right (R) lower limb, as well as for all muscles pooled together (total, T) are reported. Research participants’ identification, stimulation amplitude, frequency and electrode configurations (cathodes in black, anodes in red, and inactive in white) are also reported.

We then fed the prediction algorithm with a total of 48 assisted standing events performed by 6 individuals while different stimulation parameters were tested to search for optimal stand-scES parameters (Supplemental Fig. 3). The prediction algorithm correctly labeled as “assisted” (i.e. score between 0 and 0.5) 95.8% of the standing events considered (Supplemental Table 3). More importantly, its ranking scores varied substantially among stimulation parameters applied and investigated muscles. For example, participant A68 tested 9 different sets of scES parameters during the monitored standing session, obtaining average prediction scores ranging between 0.14 and 0.49 (Fig. 8a; Supplemental Table 3). In particular, during the standing attempt characterized by the lower score, only R IL and TA muscles showed EMG activity characteristics closer to independent standing. On the other hand, the standing attempt with the higher score was characterized by independent standing-like EMG characteristics of several muscles (i.e. posterior thigh muscles and anterior muscles of the left lower limb). Also, EMG activity score of bilateral plantar flexor muscles was low in both standing conditions.

We then exemplified that the proposed prediction algorithm can be used also for ranking the effectiveness of EMG activity collected during standing with different amount of external assistance. For instance, it correctly labeled two standing events as “independent”, and suggested that independent standing can be achieved even when the activation characteristics of few muscles are ranked as “assisted” (Fig. 8b). Also, when the algorithm is trained with the proper data set (data collected during standing with one lower limb assisted and the contralateral one generating independent extension, and during independent standing), it can rank the effectiveness of EMG activity generated by the lower limb assisted for knee extension (i.e. right side) while the other leg maintained independent extension (left side) (Fig. 8c).

## Discussion

In this study, we developed a novel data processing framework for EMG activity promoted by spinal cord epidural stimulation during standing in individuals with severe SCI. This approach allowed us to uncover physiological characteristics of neuromuscular activation resulting in independent standing with self-assistance for balance. Additionally, we showed that, for each investigated muscle, the proposed machine learning algorithm can rank the effectiveness of EMG activity generated for standing. We discuss the implications of these findings in the context of mechanisms of motor pattern generation, and for the support this framework can provide during the selection of scES parameters, suggesting that it may contribute to facilitate the clinical translation of scES for standing motor rehabilitation.

Frequency-domain EMG features have been widely considered to study central motor control strategies during voluntary muscle activation^15–19^, and the more recent development of technology for decomposing surface EMG signals has resulted in further insights on this topic^20–22^. On the other hand, EMG spectral features have been substantially neglected when the generation of activation patterns is promoted by scES. Gerasimenko and colleagues proposed a qualitative interpretation of spectral analysis (by FFT) performed on EMG signals collected from flexor and extensor muscles during stepping with scES^23^. In particular, they suggested that the dominant spectral peaks related to the stimulation frequency and its harmonics observed during the extension phase in extensor muscles reflected a predominance of monosynaptic-evoked responses. Conversely, the lack of consistent dominant peaks detected from the tibialis anterior muscle during the flexion phase of the gait cycle was interpreted as a predominance of polysynaptic-evoked responses. It is plausible that the marked dominant FFT spectral peaks related to the epidural stimulation frequency (i.e. Fig. 2a; Gerasimenko *et al*.^23^) have been often interpreted as features without relevant physiological meaning, thus discouraging further efforts aimed at quantifying scES-promoted EMG spectral parameters. Our approach was initially focused on understanding which spectral analysis method is more effective for identifying frequency-domain EMG features that characterize standing promoted by scES. This is important because, for example, FFT presents some intrinsic limitations such as poor time resolution, assuming the stationarity of EMG signal, and being unable to localize frequency content of the signal in the time domain, which may result in insufficient representation of the frequency content of scES-promoted muscle activation. Our results suggest that CWT is a spectral analysis method that can provide relevant frequency content not related to scES frequency (Fig. 2) as well as features resulting in the most accurate classification of assisted and independent standing (Fig. 3). This may be due to its high time and frequency resolution by decomposing the signal using numerous multi-resolution wavelets^24,25^, which leads to an accurate characterization of the short time component within non-stationary signals^26^. Conversely, the resolution of STFT in time and frequency domain depends on the selected window size: longer window size increases the frequency resolution but impairs time resolution, which is not ideal for non-stationary signals like EMG^27^.

To date, little is known about the characteristics of scES-promoted muscle activation resulting in the recovery of independent standing with self-assistance for balance after clinically motor complete SCI. We previously observed that the alternation between EMG bursts and negligible EMG activity (i.e. Fig. 1c, bottom) results in poor standing pattern and the need of external assistance^6^. Conversely, overall continuous (i.e. non-rhythmic) co-activation of several lower limb muscles was demonstrated when motor complete SCI individuals were able to maintain independent lower limb extension using spinal cord stimulation^6,7,13,28,29^. In this study, we identified additional EMG features that can discriminate the effectiveness of EMG activity for standing beyond the mere variability of the EMG pattern over time. In particular, independent standing events were promoted by EMG activity characterized by lower median frequency, lower variability of median frequency, lower variability of instantaneous maximum power as well as higher total power as compared to assisted standing (Figs 4–6; Supplemental Fig. 1). It is worth noting that the frequency-domain features can differentiate assisted and independent standing when the raw EMG signals are both overall continuous and demonstrate similar amplitude (Figs 4 and 7), and also when the same exact stimulation parameters are applied to the same individual resulting in different standing ability (Fig. 6; Supplemental Fig. 1). We then observed that the higher median frequency and higher variability of median frequency detected within overall continuous activation patterns during assisted standing reflect, at least partially, the sharper peaks of evoked responses, which carry more power at higher frequencies (Fig. 7). On the other hand, the smoother peaks of evoked responses detected during independent standing do not induce relevant increments in instantaneous median frequency. Partial desynchronization of motor units and/or greater involvement of polysynaptic responses, among others, may explain the smoother peaks of evoked responses detected during independent standing. Further studies involving the application of multi-channel surface EMG and dedicated signal processing may be useful for assessing the concurrent activity of many different motor units and investigating their firing pattern^30^. A limit of the present study is that it was not design to investigate how different stimulation parameters and activity-based training contributed to the activation pattern characteristics resulting in assisted or independent standing. Another limitation of the proposed framework is that it does not discriminate sufficiently between standing conditions with hips assisted and hips independent while the knees maintain independent extension (Supplemental Fig. 2; Supplemental Table 2). This may be due, at least partially, to the fact that trunk muscles contributing to hip joint control were not considered for analysis because of the presence of scES artifacts.

Presently, the prevailing view is that scES facilitates motor pattern generation by recruiting primarily large myelinated fibers associated with somatosensory information, and particularly with proprioceptive and cutaneous feedback circuits, at their entry into the spinal cord as well as along the longitudinal portions of the fiber trajectories, altering the excitability of lumbosacral spinal circuits^3–5,9–12,31–35^. This more functional excitability state, in turn, enables the spinal circuitry to use somatosensory information and residual supraspinal input as sources of control for generating motor patterns appropriate for standing and stepping. It is also important to consider that stimulation parameters play a crucial role in determining extent and proportion of the modulation of sensory-motor pathways impacted by scES^36^. For example, previous studies proposed that different stimulation frequencies may access different inhibitory and/or excitatory pathways within the spinal circuitry^37^, and that higher stimulation frequencies may promote a progressive integration of additional afferent inputs through the greater involvement of interneurons^13,38,39^. While we attempt to select higher stimulation frequencies to promote the integration of afferent and residual supraspinal input through the greater involvement of interneurons and to promote more physiological (i.e. non-pulsatile) muscle contraction, this approach may also result in “bursting” EMG patterns not effective for standing (Figs 1c and 4a). This is conceivably due to the fact that these higher frequencies are applied in combination with stimulation sites and amplitudes that result in the reconfiguration of the interneuronal network (via presynaptic and synaptic mechanisms) to favor the generation of “bursting, locomotor-like” EMG patterns^37^. Because of the important role of stimulation frequency in contributing to the characteristics of activation pattern generated, an effort was devoted to understand whether the differences in EMG features observed in the present study between assisted and independent standing, and particularly the frequency-domain features, were associated with the application of different scES frequencies. Interestingly, the average stimulation frequencies delivered during assisted and independent standing were very similar (see description of Fig. 5 and Supplemental Fig. 2 in Results). Moreover, consistent differences in EMG features were also observed between the one lower limb achieving independent extension and the contralateral lower limb requiring external assistance, while the same spinal cord stimulation was applied (Fig. 6). Taken together, these findings further suggest that the characteristics of muscle activation result from the complex interaction among the stimulation parameters applied, the somatosensory information as well as any residual supraspinal input integrated by the spinal circuitry^3,8,14^, and the characteristics of its extensive, individual-specific reorganization after SCI^40,41^.

The integration of novel CWT-derived features with EMG total power and pattern variability enabled the proposed machine learning (KNN) algorithm to accurately classify assisted and independent standing (Fig. 3). It is interesting to note that the higher classification accuracy was achieved when considering medial gastrocnemius (96.3%) and tibialis anterior (97.5%), two primary ankle muscles. This may be due to the fact that plantar flexor muscles play a significant role in controlling and stabilizing the body during bipedal quiet standing^42,43^, and that their net mechanical output is also influenced by their antagonist (TA). On the other hand, the lower classification accuracy (92.2%) resulted from the analysis of EMG collected from iliopsoas, a non-antigravity muscle that was monitored via fine-wire electrodes, which presented a greater intrinsic placement variability compared to the surface electrodes used for all other muscles. However, we took advantage of this overall high classification accuracy to develop a prediction algorithm capable of ranking the activation effectiveness of the investigated muscles for standing (Fig. 8; Supplemental Table 3). This approach results in a quasi-real time feedback on the effectiveness of scES-promoted muscle activation for standing, which can support researchers and clinicians during the process of selection of stimulation parameters. For example, the data reported in Fig. 8a suggests that left and right plantar flexors presented poor activation with both sets of stimulation parameters, being one of the possible factors limiting the achievement of independent standing. While the present framework does not propose the specific stimulation parameters adjustment for optimizing muscle activation, it can substantially improve the application of guidelines previously suggested for this task^13^. For instance, information on the individualized map of motor pools activation^44,45^ may be retrieved and used to determine the electrode field of an additional interleaving program aimed at targeting primarily the location of the spinal circuitry related to plantar flexors. Then, a much smaller cohort of cathode-anode combinations as well as amplitude and frequency values can be tested, thus increasing the probability of achieving an improved activation pattern in a reduced amount of time. This is of particular interest considering that over 40 million different combinations of electrode configurations are potentially available when using a 16-electrode array, and that minor adjustments in the electrode configuration may or may not affect significantly standing motor pattern^13^. The second important contribution of the proposed framework is that it can suggest which of the tested set of stimulation parameters promotes activation patterns more effective for standing. This can be relevant when different sets of parameters result in the same need of external assistance (i.e. total score of Fig. 8a,c; Supplemental Table 3), and the decision on which parameters to apply for stand training should be made.

In conclusion, we have demonstrated that the proposed data analysis framework can characterize time- and frequency-domain EMG features resulting in the recovery of independent standing with self-assistance for balance in individuals with motor complete SCI using spinal cord epidural stimulation. This allowed us to develop a machine learning algorithm capable of ranking the effectiveness of muscle-specific activation for standing, which may facilitate the process of selection of stimulation parameters for standing motor rehabilitation. Future studies should be aimed at investigating the effects of stimulation parameters modulation on the EMG features related to standing ability. Also, the application of a similar framework on EMG activity collected during stepping with epidural stimulation may provide novel insights on mechanisms of motor pattern generation and selection of epidural stimulation parameters.

## Methods

### Participants

Eleven individuals with chronic, clinically motor complete or sensory and motor complete SCI individuals are included in this study (Table 1). The research participants signed an informed consent for lumbosacral spinal cord epidural stimulator implantation, stimulation, activity-based training and physiological monitoring studies, which were conducted according to the standards set by the Declaration of Helsinki, and were approved by the University of Louisville Institutional Review Board (ClinicalTrials.gov identifiers NCT02037620 and NCT02339233). Prior to epidural stimulator implantation, the International Standards for Neurological Classification of Spinal Cord Injury^46^ was used for classifying the injury using the ASIA (American Spinal Injury Association) Impairment Scale (AIS). The research participants were implanted with a spinal cord epidural stimulation unit over 8 years (2009 to 2017), and were enrolled into interventional studies focused on either the facilitation of standing and stepping or the recovery of cardiovascular function. The research participants and other persons appearing in figures and supplemental videos included in this paper also gave written informed consent and granted full permission for their image to be used in publication online.

**Table 1:** Clinical characteristics of the research participants.

ID	Age (yrs)	Sex	Duration of Injury (Yrs)	Neurological Level	AIS Grade	AIS Score						Anal sensation	Anal contraction	Intervention
						Sensory (T10 - S5, core out of 24)				Motor (lower extremity)				
						L LT	L PP	R LT	R PP	L	R			
B13	33	M	4.2	C7	B	10	10	10	8	0	0	Yes	No	Motor #1
B07	24	M	3.4	T2	B	15	11	18	10	0	0	Yes	No	Motor #1
A45	24	M	2.2	T4	A	0	0	0	0	0	0	No	No	Motor #1
A53	27	M	2.3	T4	A	0	0	0	0	0	0	No	No	Motor #1
B23	32	M	3.3	C5	B	8	0	10	0	0	0	Yes	No	Motor #2
A59	26	M	2.5	T4	A	0	0	0	0	0	0	No	No	Motor #2
B30	22	F	3.3	T1	B	17	5	17	9	0	0	Yes	No	Motor #2
A60	23	M	3.1	T4	A	0	0	0	0	0	0	No	No	Motor #2
A68	35	M	3.8	C4	A	0	0	0	0	0	0	No	No	Cardiovascular
A41	24	M	7.2	C4	A	0	0	0	0	0	0	No	No	Cardiovascular
B21	31	M	7.0	C4	B	1	1	0	0	0	0	Yes	No	Cardiovascular

Sensory score was designated by light-touch (LT) and pinprick (PP) of the left (L) and right (R) lower limb, below the level of injury. Neurological level: neurological level of the lesion; AIS: American Spinal Injury Association (ASIA) Impairment Scale. Each research participant was enrolled in an interventional study focused on either the facilitation of standing and stepping (Motor #1 and Motor #2) or the recovery of cardiovascular function (Cardiovascular).

### Surgical implantation of electrode array and stimulator

The epidural spinal cord stimulation unit (Medtronic, RestoreAdvanced) and the 16-electrode array (Medtronic, 5-6-5 Specify) were surgically implanted in the eleven research participants. The electrode array was positioned over the midline of the exposed dura, in correspondence of spinal segments L1-S1/S2 (Supplemental Fig. 3)^5,47^. EMG recordings from leg muscles were obtained intraoperatively during spinal stimulation at 2 Hz using midline, left and right electrode pairs in order to localize the optimal placement of the array. The wire leads were then internalized and tunneled subcutaneously to the abdomen and connected to the neurostimulator.

### Experimental procedures

Experimental sessions devoted to the assessment of motor patterns generated during standing were performed over ground in a full bodyweight bearing condition, using a custom-designed standing apparatus. This standing apparatus is comprised of horizontal bars anterior and lateral to the individual that were used for upper extremity support and balance assistance as needed. Mirrors were placed in front of the participants and laterally to them, in order to provide visual feedback on their body position. Four individuals (B23, A59, B30, A60) performed standing also using a walker that was fixed to a wider aluminum frame base, a regular walker, or holding the hands of a trainer (hand-hold). Research participants always self-assisted balance control using their upper limbs during the standing events considered in this study.

scES was applied while the participant was seated. The sit to stand transition was performed with the research participants using their upper limbs to partially pull themselves into a standing position, and trainers positioned at the pelvis and knees manually assisting as needed the transition. If needed, research participants with higher level of injury and limited upper limb function were also assisted by trainers at the axillary triangle during the sit to stand transition.

When a stable standing position was achieved, if the knees or hips flexed beyond the normal standing posture, external assistance was provided at the knees distal to the patella to promote extension, and at the hips below the iliac crest to promote hip extension and anterior tilt. In particular, external facilitation was provided either manually by a trainer or by elastic cords, which were attached between the two vertical bars of the standing apparatus.

### Selection of scES parameters for standing

The subset of scES parameters tested to facilitate standing were selected following dedicated guidelines^13^, which are based on the literature as well as on previous assessments performed on the same research participants in supine position. Stimulation site and electrodes configuration have important implications for both topographical and functional organization of the activation pattern facilitated by scES. For example, we defined electrode fields that were more focused on the caudal portion of the electrode array to increase the excitability of distal muscles’ motoneuron pools, or selected electrode fields that were more extended toward the rostral portion of the array to increase the excitability of proximal muscles’ motoneuron pools^44^. Additionally, we initially positioned cathodes (active electrode) caudally, and more caudally than anodes, as this was shown to possibly promote better motor patterns characteristic of standing behavior in clinically motor complete SCI individuals while lying supine and standing^13,37^. Also, in case of activation differences between left and right lower limb, active electrodes were unbalanced between lateral columns of the electrode array, as the lateral placement of the epidural stimulation electrodes with respect to the spinal cord midline was shown to promote motor responses in muscles ipsilateral to the stimulation^9^. Furthermore, we adjusted cathodes position in order to target primarily extensors muscle groups according to the individualized map of motor pools activation. This was determined during previous assessments of muscle activation responses to different localized, two-electrode configurations using 2 Hz stimulation frequency, with the research participants in supine position (similarly to what has been previously reported^44^).

Epidural stimulation was initially delivered at a near-motor threshold stimulation amplitude that did not elicit directly lower limb movements in sitting, as the goal was to allow sensory information (and possibly residual descending input) to modulate the motor pattern. Stimulation amplitude and frequency were then synergistically modulated during standing in order to identify the higher stimulation frequency that elicited a continuous (non-rhythmic) EMG pattern effective to bear body weight, because higher stimulation frequencies may favor the integration of afferent and residual supraspinal input through the greater involvement of interneurons^38,39^ and result in a more physiological (i.e. non-pulsatile) muscle contraction. These guidelines were also applied to multiple interleaving programs (i.e. Figs 1b and 4), which allow the access of different locations of the spinal circuitry with different stimulation intensities and frequencies. Each research participant underwent one or two experimental sessions aimed at selecting appropriate scES parameters for standing prior to the beginning of stand training. Stimulation parameters were also adjusted throughout stand training. In particular, dedicated sessions were performed approximately every 2–4 weeks to monitor motor behavior and lower limb EMG activity while testing different stimulation parameters to contribute to their selection.

### Activity-based interventions

The standing experimental sessions considered for the present study were always performed using scES, and were carried out after scES implantation and prior to any training as well as after the different interventions defined for each of the three study groups, which are briefly described here below. All activity-based training protocols were always performed with scES optimized for the task that was practiced.

*Motor #1* (Described by Rejc and colleagues^6^). Research participants underwent 81 ± 1 sessions of full weight-bearing stand training (1 hour of standing, five sessions per week). Stand training was performed using the custom-designed standing apparatus previously described. Participants were encouraged to stand for as long as possible throughout the training session, with the goal of standing for 60 min with the least amount of assistance. Seated resting periods occurred when requested by the individuals. Following the completion of stand training and respective experimental sessions, the research participants performed 81 ± 2 sessions of step training with body weight support (Innoventor, St. Louis, MO) on a treadmill (1 hour, five sessions per week). Body weight support, stepping speed and bouts duration were adapted to each individual to obtain appropriate stepping kinematics. Following a stepping bout, participants were encouraged to maintain standing. The research participants were also encouraged to practiced voluntary trunk and lower extremity movements with scES 5 days a week (1 hour per session).

*Motor #2* (Described by Angeli and colleagues^3^). The initial portion of the training protocol (81 ± 6 sessions) consisted of one 1-hour training session per day for five days a week, and the trained motor task (standing or stepping) was alternated every session. During the second portion of the training protocol (79 ± 6 sessions), one supplementary training session was added every two weeks to the weekly schedule, to result in two training sessions per day. The research participants were encouraged to volitionally contribute to the motor pattern generation during training. Research participants were also encouraged to practice voluntary trunk and lower extremity movements with scES 5 days a week (1 hour per session).

*Cardiovascular* This study protocol included three different interventions, which were performed in sequential order and were cumulative. Research participants presenting with persistent low resting blood pressure initially completed 83 ± 3 two-hour sessions of daily scES aimed at increasing systolic blood pressure within 105 to 120 mm Hg, as reported by Harkema and colleagues^48^. In addition to this task, they subsequently performed approximately 80 training sessions of voluntary trunk and lower extremity movements practice (5 days a week, 1 hour per session). Following the completion of voluntary movements training, research participants also included stand training in their daily activities. In particular, research participants completed 83 ± 1 stand training sessions (5 days a week, 1 hour of standing per session).

### Data acquisition

EMG, ground reaction forces and kinematics data were recorded at 2000 Hz using a custom-written acquisition software (National Instruments, Austin, TX). EMG activity of right (R) and left (L) gluteus maximus (GL), medial hamstring (MH), rectus femoris (RF), vastus lateralis (VL), tibialis anterior (TA), medial gastrocnemius (MG) and soleus (SOL) was recorded by means of bipolar surface electrodes with fixed inter-electrode distance^5^. Bilateral EMG from the iliopsoas (IL) was recorded with fine-wire electrodes. Two surface electrodes were placed symmetrically lateral to the electrode array incision site over the paraspinal muscles in order to record the stimulation artefacts, which were used as indicators of the stimulation onset (time points when the stimulus pulses were applied). Lower limb joint angles were acquired using a high-speed optical motion capture system (Motion Analysis, Santa Rosa, CA). Ground reaction forces were collected using a high-resolution pressure sensing mat (HR mat system, TEKSCAN, Boston, MA) or force platforms (Kistler Holding AG, Winterthur, Switzerland).

### Data analysis

Each standing event considered for analysis was characterized by consistent external assistance and stimulation parameters for a duration ranging between 40 and 70 seconds; the initial and final 5 seconds of each event were not considered for analysis. Each event was labeled as follow, based on whether hips and knees needed external assistance for maintaining standing or achieved independent extension: hips and knees assisted (assisted standing); hips assisted and knees independent; hips and knees independent (independent standing); one knee assisted and the contralateral knee independent.

The EMG processing framework consisted of several steps including spectral analysis, time- and frequency-domain features extraction, dimension reduction, classification and prediction, which are described here below.

### EMG time domain features

The EMG pattern variability was assessed by calculating the coefficient of variation (standard deviation / mean) of the EMG linear envelope obtained by filtering the rectified EMG signal through a low-pass digital filter (cutoff frequency: 4 Hz)^6^.

The EMG total power was calculated using the following equation:

1 $$P=\frac{1}{T}{\int }_{0}^{T}{|x(t)|}^{2}dt$$

where x(t) is the recorded EMG signal and T is the length of the signal.

For each examined muscle, the total power was then normalized by the maximum value detected within each participant.

### Spectral analysis

In this study, we initially applied three signal analysis methods to the scES-promoted EMG activity, with the goal of identifying the analysis method that better differentiates conditions of assisted standing and independent standing based on the spectral information provided. Fast Fourier Transform (FFT) is one of the most commonly used methods for spectral analysis of EMG signals^49^. It is characterized by high frequency resolution and poor time resolution, and cannot localize the frequency content of the signal in the time domain. Short-Time Fourier Transform (STFT) was designed to increase the time resolution of FFT by selecting a fixed-size window moving across the EMG signal^50^. Finally, Continuous Wavelet Transform (CWT) has been designed to effectively localize the frequency content of non-stationary signals in both time and frequency domains by using size adjustable wavelets, which do not compromise time or frequency resolutions^51–53^.

### Frequency domain features

Power spectral density (PSD) of FFT, STFT spectrogram (s(*t*, *f*) and CWT scalogram (*p*(*f*, *t*)) (using Morlet wavelet, ${\psi }_{f,t}(\tau )$) was calculated as reported in Eqs 2 to 4, respectively.

2 $$\begin{array}{c}FFT(f)=\int x(\tau )\exp (-j2\pi f\tau )d\tau ,\\ \,PSD(f)={|FFT(f)|}^{2}\end{array}$$

3 $$\begin{array}{c}STFT(t,\,f)=\int {w}^{\ast }(\tau -t)x(\tau )\exp (-j2\pi f\tau )d\tau ,\\ \,{\rm{STFT}}\,{\rm{Spectrogram}}:\,s(t,\,f)={|STFT(t,f)|}^{2}\end{array}$$

4 $$\begin{array}{c}CWT(f,\,t)=\int x(\tau ){\psi }_{f,\,t}^{\ast }(\tau )d\tau \\ \,{\rm{Morlet}}\,{\rm{wavelet}}:{\psi }_{f,t}(\tau )=\,\frac{1}{\sqrt{{f}_{0}/f}}\psi (\tau -t/({f}_{0}/f))\\ \,{\rm{Wavelet}}\,{\rm{scalogram}}:\,p(f,\,t)={|CWT(f,t)|}^{2}\end{array}$$

where *f*_0_ is the sampling frequency (2 kHz).

The STFT window size was selected at 0.3 seconds to increase the time resolution of FFT while minimally compromising the frequency resolution.

Mean frequency, median frequency, dominant frequency, and maximum power are the physiologically relevant features that were extracted from FFT output.

As for STFT and CWT, instantaneous values of mean frequency (IMNF), median frequency (IMDF), dominant frequency (F_max_(t)) and maximum power (P_max_(t)) were initially calculated (Eqs 5–8), and their average and standard deviation (SD) were considered as features for further analysis. In particular, EMG maximum power variability was assessed by calculating its coefficient of variation (SD/mean).

5 $$IMNF(t)=\frac{{\sum }_{j=1}^{M}\,{f}_{j}p({f}_{j},\,t)}{{\sum }_{j=1}^{M}\,p({f}_{j},\,t)}$$

6 $${\sum }_{j=1}^{IMDF(t)}p({f}_{j},\,t)={\sum }_{j=IMDF(t)}^{M}p({f}_{j},\,t)$$

7 $${F}_{max}(t)=argma{x}_{f}(p(f,\,t))$$

8 $${P}_{max}(t)=ma{x}_{f}(p(f,\,t))$$

where *M* is the number of frequency bins.

### Classification

All the calculated EMG feature values (predictors) were normalized to their maximum to remove the effects of their units in the classification step. The non-negative matrix factorization (NNMF) algorithm was applied to the normalized measurements for dimensionality reduction^54^ and the output values were logarithmically transformed in order to stabilize the variance^55^. We then performed preliminary analysis to determine which classification method resulted in the highest accuracy for classifying conditions of assisted standing versus independent standing based on the EMG features herein considered. In particular, K-nearest neighbor (KNN)^56^ performed better than Naïve Bayes^57^, binary Support Vector Machine^58^, and ensemble decision trees^59^; therefore, KNN was the classification method applied in the present study.

The KNN classifier includes several parameters that need to be adjusted in order to achieve its best classification performance. These parameters include number of neighbors, distance measures, distance weights and standardization (centering and scaling the predictors). In order to find the optimized parameters for the classifier, the Bayesian optimization algorithm was used^60^. The objective function for the optimization is log (1 + Cross Validation Loss). The Cross Validation Loss is the ratio of misclassified observations during the cross validation step. The classification accuracy is calculated using 10-fold cross validation method and calculated as a percentage value of 1- Cross Validation Loss^57^. The KNN classifier with the parameter optimization algorithm and the cross validation step were iterated 10 times and the average accuracy values and the 95% confidence intervals are reported.

### Prediction

All calculated EMG feature vectors (time- and CWT-derived features) that we included in the classification step were then used as a training dataset for the prediction part of the framework. A trained model is defined as a model that has captured the patterns in the training dataset. Based on these learnt patterns, the trained model can predict the class label (i.e. assisted or independent standing) for new observations that were not included in the training dataset. For this part of the study, we developed KNN models that are trained for each investigated muscle pair (i.e. left and right soleus) on three data sets related to the following different external assistance for standing: (*i*) hips and knees assisted *vs* hips and knees independent; (*ii*) one knee assisted *vs* hips and knees independent; (*iii*) hips assisted and knees independent *vs* hips and knees independent. The models related to the first data set were then used to predict the class labels for the prediction dataset, which includes assisted standing events collected from 6 research participants during experimental sessions aimed at testing the effectiveness of different scES parameters for standing. We also exemplified the application of models related to the second data set. The output of the prediction step is a score value ranging from 0 and 1, which is the posterior probability $\,P(C|{X}_{new})$ of “independent standing” class *C* given a new observation *X*_*new*_ (Eq. 9).

9 $$P(C|{X}_{new})=\,\frac{{\sum }_{i=1}^{K}W({X}_{i}){1}_{{X}_{i}=C}}{{\sum }_{i=1}^{k}W({X}_{i})}$$

Where *K* is the number of nearest neighbors to *X*_*new*_, *X*_*i*_ is the i^th^ nearest neighbor, *W*(*X*_*i*_) is the weight of *X*_*i*_ which is the distance value from *X*_*new*_ and normalized based on the class prior probability, i.e. the frequency of the number of observations in one class in the training dataset. The ${1}_{{X}_{i}=C}$ function returns 1 if observation *X*_*i*_ belongs to class C and 0 otherwise^61^.

For each muscle, score values equal or less than 0.5 assign the given observation to the “assisted standing” class label, while values greater than 0.5 assign the observation to the “independent standing” class label. The number of neighbors for the prediction task is set to *K* = 5; this keeps the classification accuracy high for all muscle pairs and allows comparison of the prediction scores between KNN models. All EMG analysis steps are performed using MATLAB R2017b software and its Statistics and Machine Learning Toolbox.

### Statistical analysis

Statistical analysis was performed using GraphPad Prism (version 5.00 for Windows, GraphPad Software, San Diego, California, USA). A p value < 0.05 was considered statistically significant. The distribution of quantitative EMG variables was tested for normality using the Kolmogorov–Smirnov test, and the parametric or non-parametric tests reported below were applied accordingly. The effect of external assistance for standing on the EMG features considered (total power, pattern variability, maximum power variability, median frequency, median frequency SD) was tested on all muscles investigated with surface EMG pooled together (left and right SOL, MG, TA, MH, VL, RF, GL), on primary extensor muscles (left and right SOL, MG, VL, RF, GL), and on primary flexor muscles (TA, MH). Additionally, we tested whether the stimulation frequency applied was significantly different among standing conditions with different amount of external assistance. In particular, paired comparisons between standing conditions of hips assisted – knees assisted and hips assisted – knees independent (subjects number = 8) were performed by Wilcoxon test. Also, comparisons among standing with hips assisted – knees assisted, hips assisted – knees independent, and hips independent – knees independent (subjects number = 5) were performed by either Repeated Measures Anova (and following multiple comparisons by Bonferroni’s post hoc test) or by Friedman Test (and following multiple comparisons by Dunn’s post hoc test). Finally, when one lower limb (i.e. left side) achieved independent extension while the contralateral limb (i.e. right side) required external assistance for knee extension, paired comparisons (subjects number = 7) between the assisted and independent side were performed by Wilcoxon test.

## Supplementary information

Supplementary information

Supplemental Tab. 3

Supplemental Video 1

Supplemental Video 2

## Data Availability

Data that support the findings and software developed for the data analysis will be made available through material transfer agreement upon request.
