# Supplementary material for: Neurophysiological markers predicting recovery of standing in humans with chronic motor complete spinal cord injury
Source: Sci Rep. 2019 Oct 9;9:14474. doi: 10.1038/s41598-019-50938-y (PMC6785550; doi:10.1038/s41598-019-50938-y)
Supplement: Supplementary file 1 — Supplementary information [file 41598_2019_50938_MOESM1_ESM.docx]

**Neurophysiological markers predicting recovery of standing in humans with chronic motor complete spinal cord injury**

Samineh Mesbah^1,2,3^, Federica Gonnelli^1,4,5^, Claudia A Angeli^1,6^, Ayman El-baz^3^, Susan J Harkema^1,6,7^, Enrico Rejc^1,7*^

^1^ Kentucky Spinal Cord Injury Research Center, University of Louisville, Louisville, Kentucky,

^2^ Department of Electrical and Computer Engineering, University of Louisville, Louisville, Kentucky, USA.

^3^ Department of Bioengineering, University of Louisville, Louisville, Kentucky, USA.

^4^ Department of Medicine, University of Udine, Udine, Italy.

^5^ School of Sport Sciences, University of Udine, Udine, Italy.

^6^ Frazier Rehab Institute, Kentucky One Health, Louisville, Kentucky, USA,

^7^ Department of Neurological Surgery, University of Louisville, Louisville, Kentucky, USA,

*** Corresponding author:**

Enrico Rejc, PhD

Kentucky Spinal Cord Injury Research Center

University of Louisville

Frazier Rehab Institute

220 Abraham Flexner Way

Louisville, KY 40202

Tel. 502-581-8747

Email: [e](mailto:SusanHarkema@KentuckyOneHealth.org)nrico.rejc@louisville.edu

**Supplemental Information**


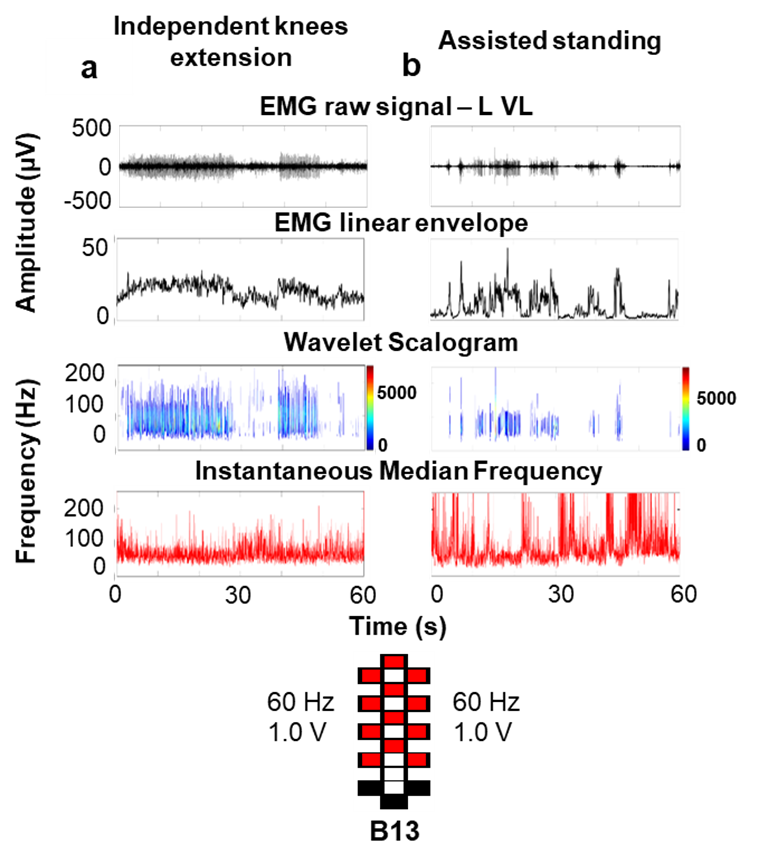


**Supplemental Fig. 1. EMG-time and –frequency features characterizing exemplary standing activation patterns promoted by the same stimulation parameters.** EMG activity was recorded from the left vastus lateralis (L VL) of participant B13 during standing with self-assistance for balance and independent knees extension (**a**) or with external assistance for knees extension (**b**). For both standing conditions, the EMG linear envelope, time-frequency power distribution of the signal (wavelet scalogram), and instantaneous median frequency are calculated from the plotted raw EMG. The wavelet scalogram is presented as contour plots, the power values of which are represented as colormaps, with the color scale showing the range of power values. Stimulation amplitude, frequency and electrode configuration (cathodes in black, anodes in red, and inactive in white) are reported. EMG activity reported in Panel (**a**) was collected after 80 sessions of stand training with epidural stimulation, and EMG activity reported in Panel (**b**) after subsequent 80 sessions of step training with epidural stimulation (see “Activity-based interventions” in the Methods for details).


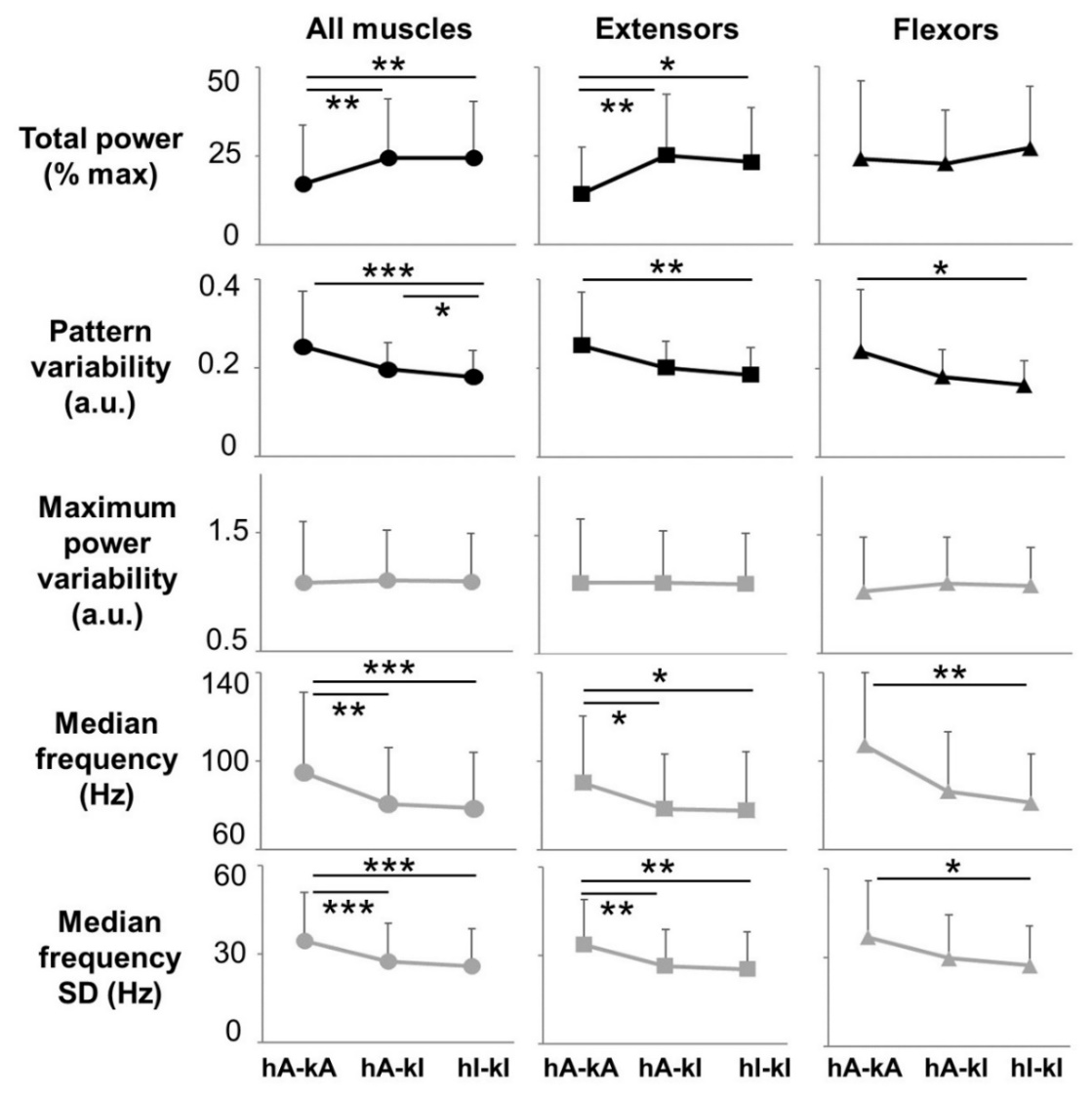


**Supplemental Fig. 2.** **Quantification of EMG-time and –frequency domain features collected during standing with different amount of external assistance.** Representative time- and frequency-domain EMG features collected during standing with external assistance for hips and knees extension (hA-kA), during standing with hips assisted and independent knees extension (hA-kI), and during standing with independent hips and knees extension (hI-kI). EMG features values were averaged among research participants (n = 5) and among all investigated muscles (left and right soleus, medial gastrocnemius, vastus lateralis, rectus femoris, gluteus maximus, tibialis anterior and medial hamstring; total n = 70), primary extensor muscles (left and right soleus, medial gastrocnemius, vastus lateralis, rectus femoris, gluteus maximus; total n = 50), or primary flexor muscles (left and right tibialis anterior and medial hamstring; total n = 20). Values are expressed as mean ± standard deviation (SD). Differences were tested by either Repeated Measures Anova (and following multiple comparisons by Bonferroni’s post hoc test) or by Friedman Test (and following multiple comparisons by Dunn’s post hoc test), depending on the data distribution characteristics. * p < 0.05; ** p < 0.01; *** p < 0.001.


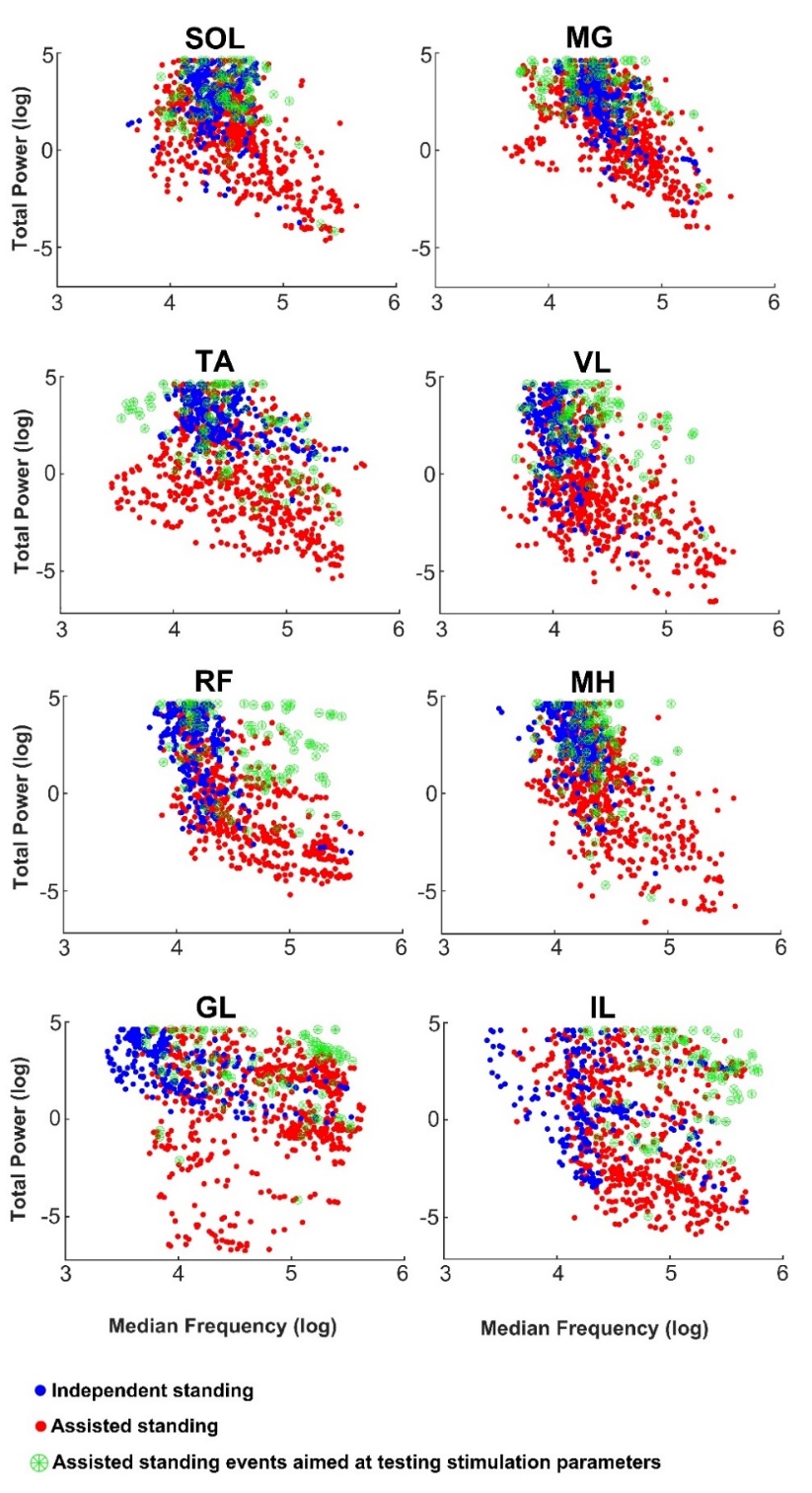


**Supplemental Fig. 3.** **Features of the EMG data sets used to train classification and prediction algorithms.** Two representative features (total power and median frequency) of the EMG data sets used to train assisted (red) and independent (blue) standing KNN models are plotted against each other for each investigated muscle. Trends of assisted and independent standing data points are overall similar across muscles, while the distribution pattern shows some inter-muscle variability (i.e. between TA and GL). EMG data collected from 6 individuals during a total of 48 standing events aimed at testing the effectiveness of different stimulation parameters (green stars) were then fed to the prediction algorithm. While all these attempts resulted in assisted standing, the related EMG data points are spread across the plots, with some of them partially overlapping independent standing data points.

**Supplemental Fig. 4.** Schematics of the 16-electrode array configuration relative to spinal cord segments^5^. The drawing is an estimation and might not be representative for each participant.

**Supplemental Tab. 1.** Number of events considered for each research participant and standing condition.

| **Participant** |  | **Hips and knees assisted (N)** | |  | **Hips assisted  knees independent (N)** | | |  | | **Hips and knees independent (N)** | |  | | **One knee assisted** | |  |
| --- | --- | --- | --- | --- | --- | --- | --- | --- | --- | --- | --- | --- | --- | --- | --- | --- |
| B13 |  | 70 | |  | 11 | | |  | | 0 | |  | | 0 | |  |
| B07 |  | 36 | |  | 10 | | |  | | 0 | |  | | 0 | |  |
| A45 |  | 13 | |  | 7 | | |  | | 10 | |  | | 6 | |  |
| A53 |  | 13 | |  | 0 | | |  | | 14 | |  | | 3 | |  |
| B23 |  | 4 | |  | 12 | | |  | | 12 | |  | | 0 | |  |
| A59 |  | 8 | |  | 5 | | |  | | 12 | |  | | 30 | |  |
| B30 |  | 1 | |  | 3 | | |  | | 59 | |  | | 2 | |  |
| A60 |  | 2 | |  | 7 | | |  | | 20 | |  | | 4 | |  |
| A68 |  | 74 | |  | 2 | | |  | | 0 | |  | | 5 | |  |
| A41 |  | 47 | |  | 0 | | |  | | 0 | |  | | 21 | |  |
| B21 |  | 48 | |  | 0 | | |  | | 0 | |  | | 0 | |  |
| **Total standing events (N)** | |  | ***316*** | | |  | ***57*** | |  | | ***127*** | |  | | ***71*** | |

**Supplemental Tab. 2.** Classification accuracy of the K-nearest neighbor models trained for each individual muscle with three different data sets related to different external assistance for standing.

|  | **Classification Accuracy (%)** | | | | | | | |  |
| --- | --- | --- | --- | --- | --- | --- | --- | --- | --- |
| **Training Dataset** | **SOL** | **MG** | **TA** | **VL** | **RF** | **MH** | **GL** | **IL** | **Average** |
| Hips and knees assisted –  Hips and knees independent | 95.8 | 96.3 | 97.5 | 95.5 | 95.4 | 95.7 | 94.4 | 92.2 | 95.3 |
| One knee assisted –  Hips and knees independent | 93.3 | 92.8 | 91.7 | 92.8 | 87.2 | 90.5 | 87.8 | 88.9 | 90.6 |
| Hips Assisted and knees independent –  Hips and knees independent | 78.8 | 82.6 | 79.9 | 78.9 | 77.9 | 81.0 | 78.3 | 85.0 | 80.3 |

**Supplemental Tab. 3.** Prediction algorithm output ranking the muscle-specific effectiveness of EMG activity for standing promoted by the tested sets of spinal cord epidural stimulation (scES) parameters, which resulted in assisted standing. Score values equal or lower than 0.5 assign the given observation to the “assisted standing” class label, while values greater than 0.5 assign the observation to the “independent standing” class label. Electrode configuration (numbers define the active electrodes referring to the 16-electrode array schematics reported in Supplemental Fig. 3; - : cathodes; + : anodes), stimulation frequency and amplitude are reported. Stimulation parameters are reported for multiple programs (P) when they were delivered sequentially.

**Supplemental video legends**

**Supplemental Video 1.** Representative sitting to standing transition and assisted standing without spinal cord epidural stimulation (scES) performed by participant A59. Also, exemplary independent standing promoted by scES; and assisted standing with scES resulting from an overall continuous activation pattern or from the alternation of EMG bursts and little activation are shown. The EMG recordings and stimulation parameters applied for each standing condition are reported in Figure 1.

**Supplemental Video 2.** Representative standing with spinal cord epidural stimulation performed by participant A45 during independent standing with self-assistance for balance, during standing with external assistance for knees extension resulting from an overall continuous activation pattern, and during standing with external assistance for hips and knees extension resulting from the alternation of EMG bursts and little activation. The EMG recordings and stimulation parameters applied for each standing condition are reported by Rejc and colleagues^6^.
